# Detection of foaling using a tail-attached device with a thermistor and tri-axial accelerometer in pregnant mares

**DOI:** 10.1371/journal.pone.0286807

**Published:** 2023-06-02

**Authors:** Takahiro Aoki, Makoto Shibata, Guilherme Violin, Shogo Higaki, Koji Yoshioka

**Affiliations:** 1 Department of Veterinary Medicine, Obihiro University of Agriculture and Veterinary Medicine, Obihiro, Hokkaido, Japan; 2 National Institute of Animal Health, National Agriculture and Food Research Organization, Tsukuba, Ibaraki, Japan; 3 Laboratory of Theriogenology, School of Veterinary Medicine, Azabu University, Sagamihara, Kanagawa, Japan; University of Agriculture Faisalabad, PAKISTAN

## Abstract

It is desirable to attend to the mare at the time of foaling in order to assist fetal delivery and prevent complications. The early detection of the onset of labor is an important issue for the equine industry. The purpose of this study was to examine the applicability of a sensor for foaling detection using the data of surface temperature (ST), roll angle (rotation about the y-axis) and y-axis (long axis of the tail) acceleration which were collected from a multimodal device attached to the ventral tail base of the mare. The data were collected every 3 minutes in 17 pregnant mares. Roll angle differences from the reference values and the mare’s posture (standing or recumbent) confirmed by video were compared and associated. Cohen’s kappa coefficient was 0.99 when the threshold was set as ± 0.3 radian in roll angle differences. This result clearly showed that the sensor data can accurately distinguish between standing and recumbent postures. The hourly sensor data with a lower ST (LST < 35.5°C), a recumbent posture determined by the roll angle, and tail-raising (TR, decline of 200 mg or more from the reference value in y-axis acceleration) was significantly higher during the last hour prepartum than 2−120 hours before parturition (P < 0.01). The accuracy of foaling detection within one hour was verified using the following three indicators: LST; lying down (LD, change from standing to recumbent posture); and TR. When LST, LD and TR were individually examined, even though all indicators showed that sensitivity was 100%, the precision was 13.1%, 8.1% and 2.8%, respectively. When the data were combined as LST+LD, LST+TR, LD+TR and LST+LD+TR, detection of foaling improved, with precisions of 100%, 32.1%, 56.7% and 100%, respectively. In conclusion, the tail-attached multimodal device examined in this present study is useful for detecting foaling.

## 1. Introduction

The mare’s gestational length is about 11 months, but it can be highly variable between individuals, lasting from 320 to 360 days [1, 2]. More than 80% of foalings occur at night [3, 4]. The mare’s second stage of labor, which starts at rupture of the allantochorion and ends at the complete birth of the neonate, is shorter when compared to other livestock animals, lasting 15–20 minutes on average. If it exceeds 40 min, the percentage of foals born dead rapidly rises [5]. Equine dystocia occurs in up to 10% of all parturitions and may present a life-threatening condition for both the foal and its dam [6]. Thus, it is desirable that foaling is always attended, in order to assist in the process and prevent complications, but a night watch requires long working hours and is an extra cost to horse breeders. In this context, prediction of parturition and detection of the onset of labor are important issues for the equine industry. Over the past few decades, with the objective of reducing the enormous cost and labor force required by foaling management, many studies have been conducted to predict or detect the time of foaling. As a result, some physiological and behavioral changes have been described as signs of parturition.

Studies have reported that the dam’s body temperature decreases before parturition in many mammals, and this phenomenon is generally considered to be associated with hormonal changes, such as in progesterone levels. In cattle [7], sheep [8] and dogs [9], body temperature has been shown to be a relevant indicator of impending parturition. Several studies also reported that body temperature decreases just before foaling in mares [10–12]. However, examination of body temperature is not commonly used in the field for predicting time of foaling. Since the decrease in body temperature occurs more rapidly in mares than in other livestock animals, routine body temperature measurements 2 or 3 times a day may miss the onset of labor.

Behavioral changes that occur before parturition have been reported in various animal species. Dairy cows kept indoors show restless behavior 4 to 6 hours before giving birth, characterized by an increase in position changes from standing to lying (lying bouts). Also, when kept in an individual maternity pen during labor dairy cows nearly doubled their lying bouts on the day of calving when compared with previous days [13]. In the sow, nest-building activity is at its peak between 6 and 12 hours preceding the expulsion of the first piglet [14, 15]. After entering the second stage of parturition, there was a decrease in changes in posture and duration of lying gradually increase in the sow [16]. In the ewe, walking behavior increased in the hours surrounding parturition, particularly within 1 hour before it. The number of posture changes per hour increased at 4 hours before parturition, reaching a maximum at the time of parturition [17]. Although some behavioral changes were reported in the mare before parturition [18], we could not find any studies that quantitatively examined hourly behavioral changes around parturition in the mare. Shaw *et al*. did investigate daily behavioral changes of prepartum mares during the last 2 weeks of pregnancy. This study reported a significant increase in the percentage of mares that spent the day of foaling in the sternal or recumbent positions [19].

Tail-raising (TR) behavior has been known to be associated with calving in the cow [20]. The occurrence of this behavioral sign increases approximately 6 hours before calving [20, 21]. A study with five cows using tail-mounted sensors demonstrated that TR might be a promising indicator for imminent calving [22]. The frequency of TR increased as parturition approached in mares when using a tail-mounted accelerometer [12]. However, there have been no studies in which TR behavior has been used to detect or predict foaling.

The purpose of the present study was to clarify the changes in body temperature and behavior using a multi-modal device equipped with a thermistor and tri-axial accelerometer attached to the ventral tail base and to examine the applicability of the sensor for detecting foaling in pregnant mares.

## 2. Materials and methods

### 2.1. Animals

This present study was conducted on two private farms (farm O and farm N) in Tokachi, Hokkaido, Japan. Seventeen pregnant heavy draft mares (3 primiparous and 14 multiparous mares) that foaled from February to May 2021 were included in the present study. The mean age of the mares was 7.6 years (range: 4−14 years). Only dry hay was fed *ad libitum* before foaling. The mares were housed in groups during late gestation, and were moved to individual stalls or paddocks for monitoring as parturition approached. The timing of moving mares to maternity stalls or paddocks was determined by the horse’s respective owner, based on the observation of physiological changes such as udder development and presence of milk in the teats. The mares were kept in paddocks (approximately 20m × 30m) with 3−4 mares throughout the day at farm O. The mares were put out to graze with other mares during the daytime, and were kept in individual stalls (approximately 4m × 8m) at farm N during the night. Prepartum dams were monitored and recorded with a webcam (BB-ST162A, Panasonic Connect Co., Ltd., Ginza, Tokyo, Japan). The video data was analyzed to identify posture changes (standing or lying) and TR behavior, as well as to determine the time of foaling. For some of the foalings that occurred under the supervision of a horse manager, foaling assistance was performed if the foaling process did not proceed during the second stage of labor. All protocols and procedures were approved by the Animal Care and Use Committee, Obihiro University of Agriculture and Veterinary Medicine (approval no. 20–220 and 21–25).

### 2.2. Tail-attached device

**Fig 1:**
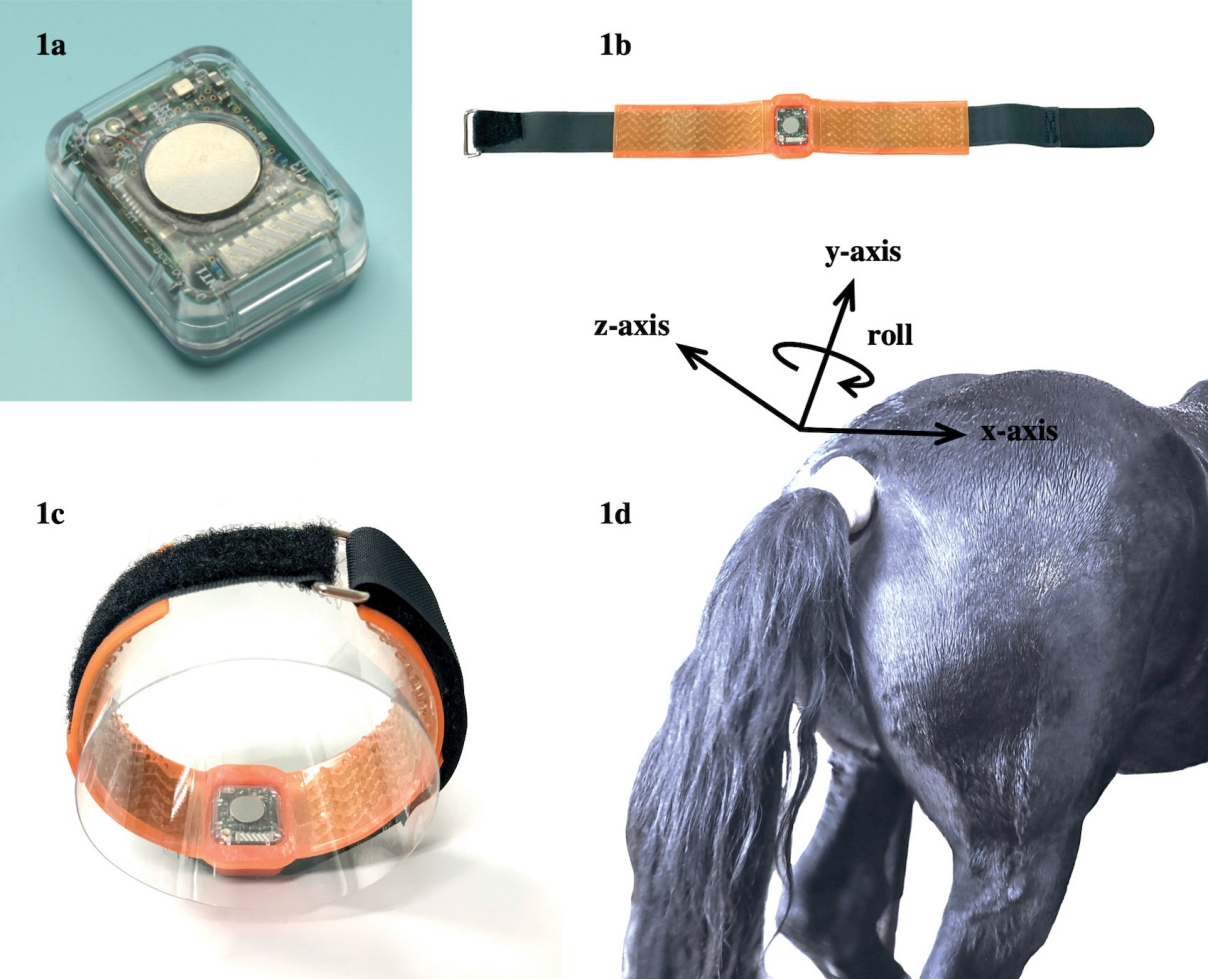
The sensor (Fig 1A) was fixed to the tail using a custom-made silicone rubber belt and a hook-and-loop fastener (Fig 1B). Fig 1C shows the sensor attached to a PET bottle shaped like a tail. The sensor attached to the tail was covered with an elastic medical bandage to stabilize its position (Fig 1D).

The ventral tail base surface temperature (ST), activity intensity, roll angle and y-axis acceleration were measured using the sensors described in previous reports [23, 24]. The dimensions of the sensors were 26.0 × 21.0 × 9.7 mm and weighed 5.8g with a battery inserted (**Fig 1A**). The thermistor of the sensor was placed on the surface of the ventral tail base, and the sensor was fixed to the tail using a custom-made silicone rubber belt and a hook-and-loop fastener (**Fig 1B and 1C**). It was also covered with an elastic medical bandage (SRPH75; NICHIBAN, Tokyo, Japan) to stabilize its position. Orientations of the x-, y-, and z-axes of the tri-axial accelerometer were lateral, proximal/distal, and dorsal/ventral to the tail, respectively (**Fig 1D**). The sensor was attached to the pregnant mare from 6.8 ± 2.9 days before the expected foaling date (defined as 335th date from the last mating) until at least one day after foaling. The tail sensor measured ST (in the range of 20 to 45°C, with 0.05°C resolution), activity intensity (in the range of 0 to 102.3, with 0.2 resolution), roll angle (rotation of the x- and z-axes about the y-axis: range of −3 to +3 rad, with 0.05-rad resolution), and y-axis acceleration (in the range of −1000 to +1000 mg, with 4 mg resolution). The activity intensity and roll angle were calculated in accordance with a previous study [23]. The y-axis acceleration has positive, zero, and negative values when the tail is down, horizontal, and raised above the horizontal, respectively. Therefore, a variation of y-axis acceleration from a reference value to a minus direction was referred as "TR index" in this present study. The reference values were defined as mean values of y-axis acceleration for 24 hours after the onset of recording. The mare’s behavior was confirmed by video at the time points determined to be a TR behavior from the sensor data. The sensor wirelessly transmitted the data to the receiver in real-time at 3-min intervals. The carrier frequency of the transceiver was 920 MHz, and the communication distance to the receiver was approximately 100 m without obstacles. The data from the sensors were automatically transmitted to a receiver and stored on a cloud server via 3G/LTE. Since the data was not stable for a while after attaching the sensor, data recording started 1 hour after the attachment.

### 2.3. Threshold for distinguishing the mare’s postures

The value of roll angle changes when changing from the standing position to the recumbent position. The reference values were defined as mean values of roll angle for 2 hours (after confirming that the mares were standing in the video) after the onset of recording. However, as for the mares whose behavior was not stable after attaching the sensor (frequently switching of posture, etc.), a reference value was calculated only after confirming by video that their behavior had stabilized and were able to stand up continuously. In this present study, changing from a reference value (standing position) to a positive value indicates lying on the left side, and changing to a negative value indicates lying on the right side. After comparing the roll angle data and the mare’s posture in the video, a threshold suitable for distinguishing the mare’s postures such as standing, sternal or lateral recumbent position was determined using Cohen’s Kappa coefficient (κ) [25]. The roll angle value closest to κ = 1.0 was determined as the optimal threshold.

### 2.4. Changes during the perinatal period

Changes in each parameter from 120 hours prepartum to 24 hours postpartum were calculated. The maximum hourly ST, hourly averaged activity intensity, total lying time per hour, total number of lying bouts per hour and hourly averaged TR index were calculated to minimize the impact of rapid momentary changes in the raw data. Since farm N had different ways to manage mares between daytime and nighttime, the data from farm N was not used to calculate the activity intensity.

### 2.5. Distribution of raw data

The hourly distribution of the raw data was plotted in order to clarify the characteristic changes that may occur just before the fetal delivery. For the characteristic parameters that may occur 1 hour before parturition, the hourly occurrence (%) was compared with the average occurrence rate (%) between 2 to 120 hours prepartum using the χ^2^ test. All statistical analyses were performed using Excel statistics (Statcel4, Oms Publications, Tokorozawa, Saitama, Japan), and P < 0.05 was regarded as statistically significant.

### 2.6. Applicability to detect the time of birth of the foal

Using the approach outlined above, it was possible to determine some characteristic parameters that may occur immediately before the time of birth of the foal. The accuracy of detecting the time of birth of the foal within 1 hour was examined using parameters such as true positive (TP), false positive (FP), false negative (FN), sensitivity and precision. If the same parameter occurred 2 or more times within 1 hour, it was counted as 1 per hour. TP was the number of mares that gave birth within 1 hour after the condition occurred. FP was the number of mares that did not give birth within 1 hour after the condition occurred. FN was the number of mares that gave birth with no condition being confirmed within 1 hour. The sensitivity and precision were calculated according to the following equations: sensitivity = TP/(TP+FN) × 100; precision = TP/(TP+FP) × 100.

## 3. Results

Background information and foaling records of the 17 mares used in this present study are shown in **Table 1**. Fifteen of the 17 mares gave birth at night (between 18:00 and 06:00 the next morning). Seven mares were assisted in the fetal delivery by managers. All 17 newly-born foals were healthy at birth with no physical abnormalities. Raw data from the sensors are shown in S1 Data.

**Table 1:** Background and foaling information of the mares used in this study.

No.	Farm	Horse name	Age	Parity	Date on which the sensor was attached to the mare	Date of foaling	Time of fetal delivery	Delivery assistance	Gestation length (days)
1	O	VIO	6	Multiparous	Apr. 14	Apr. 18	18:42	Unassisted	330
2	O	KAN	13	Multiparous	Mar. 8	Mar. 14	2:27	Unassisted	338
3	O	GOL	6	Multiparous	Mar. 31	Apr. 7	9:17	Assisted	335
4	O	SMI	9	Multiparous	Apr. 21	Apr. 23	23:51	Unassisted	328
5	O	SER	5	Multiparous	Mar. 20	Mar. 27	4:17	Unassisted	326
6	O	DEE	4	Primiparous	Apr. 21	Apr. 29	23:44	Unassisted	339
7	O	TOR	14	Multiparous	Feb. 10	Feb. 19	21:01	Assisted	346
8	O	POW	9	Multiparous	Mar. 16	Mar. 25	21:20	Unassisted	338
9	O	BLU	8	Multiparous	Apr. 6	Apr. 17	0:34	Unassisted	338
10	O	WHI	5	Multiparous	Mar. 2	Mar. 14	21:08	Unassisted	322
11	O	YUN	10	Multiparous	Apr. 15	Apr. 21	5:40	Assisted	334
12	O	YOS	8	Multiparous	Mar. 30	Apr. 3	4:18	Unassisted	328
13	O	RAS	8	Primiparous	Apr. 21	Apr. 22	6:43	Assisted	332
14	N	SHO	4	Primiparous	Feb. 10	Feb. 20	2:05	Unassisted	335
15	N	KUR	9	Multiparous	Mar. 16	Mar. 22	21:32	Assisted	338
16	N	TEI	5	Multiparous	Mar. 19	Mar.25	21:56	Assisted	337
17	N	HAM	5	Multiparous	Mar. 11	Mar. 18	4:08	Assisted	335

Gestation length was defined as the days from the date of last mating to the date of parturition.

**Fig 2:**
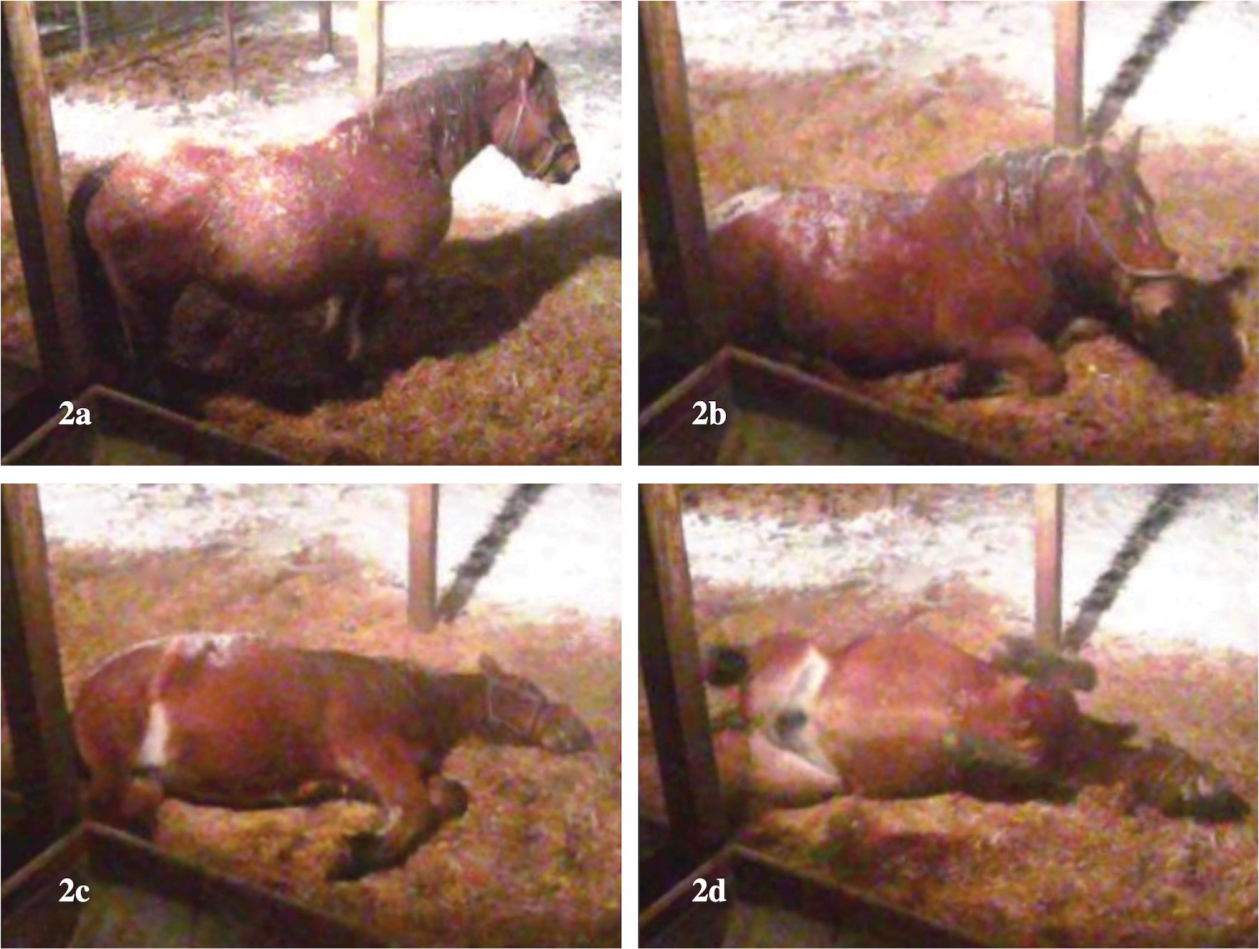
The 27,473 time points were annotated for each posture according to the mare’s actual posture confirmed in the video. 2a: standing position, 2b: sternal recumbency, 2c: lateral recumbency, 2d: dorsal recumbency (a few mares momentarily showed this position, but were recorded as recumbent position in sensor data).

**Fig 3:**
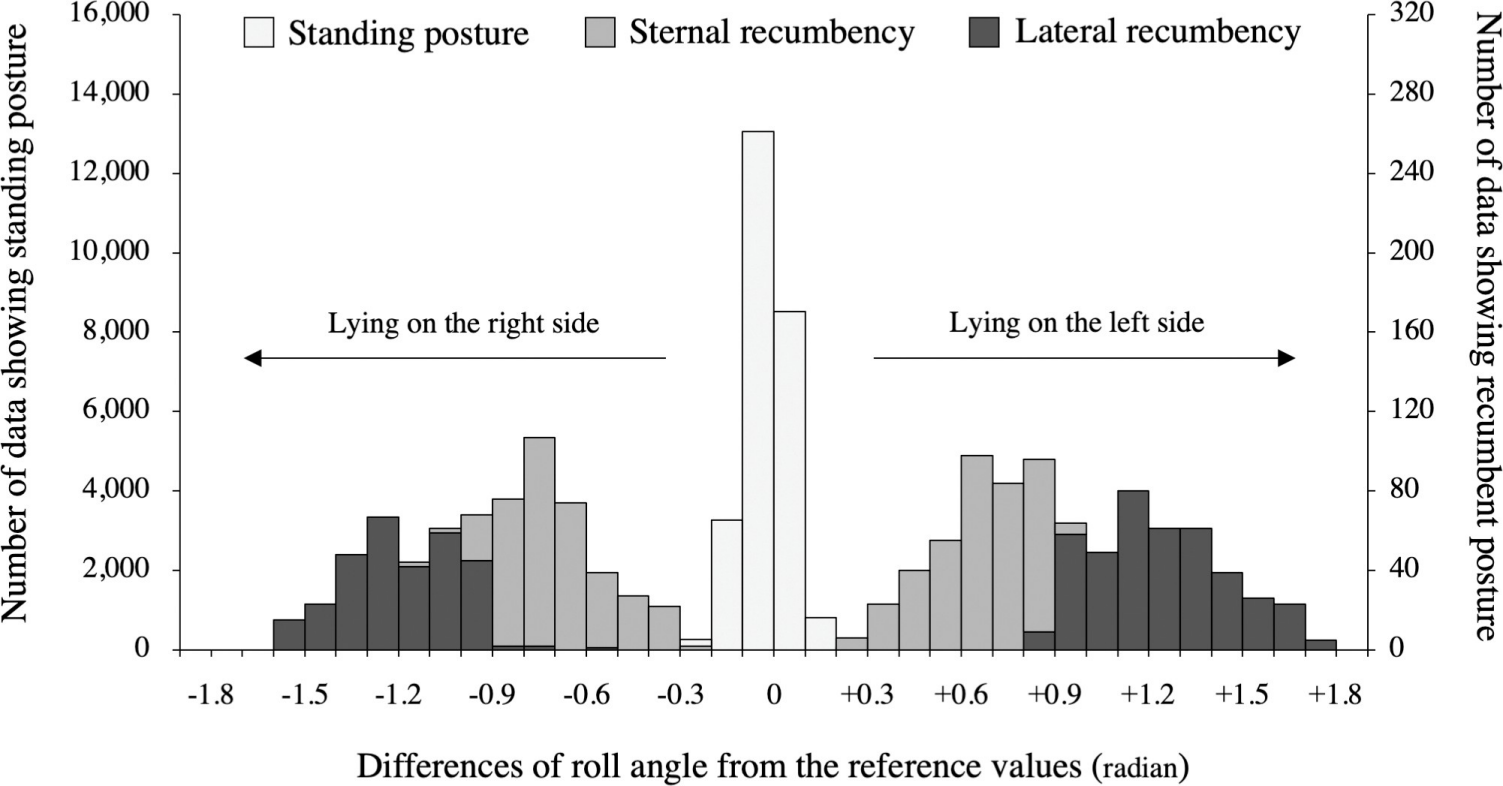
The relationship between the mare’s posture determined by the video and the roll angle data.

The 27,473 time points were annotated for each posture according to the mare’s posture confirmed by video (**Fig 2**). The relationship between the mare’s posture determined by the video and the roll angle data are shown in **Fig 3**. Cohen’s kappa coefficient between the predicted value (based on the roll angle) and the true value (based on the video analysis) for standing posture and recumbent postures (sternal and lateral recumbencies) was 0.99 when the threshold was set to ± 0.3 radian from the reference values of roll angle. Also, Cohen’s kappa coefficient for sternal and lateral recumbent posture was 0.94 when the threshold was set to ± 0.9 radian.

**Fig 4:**
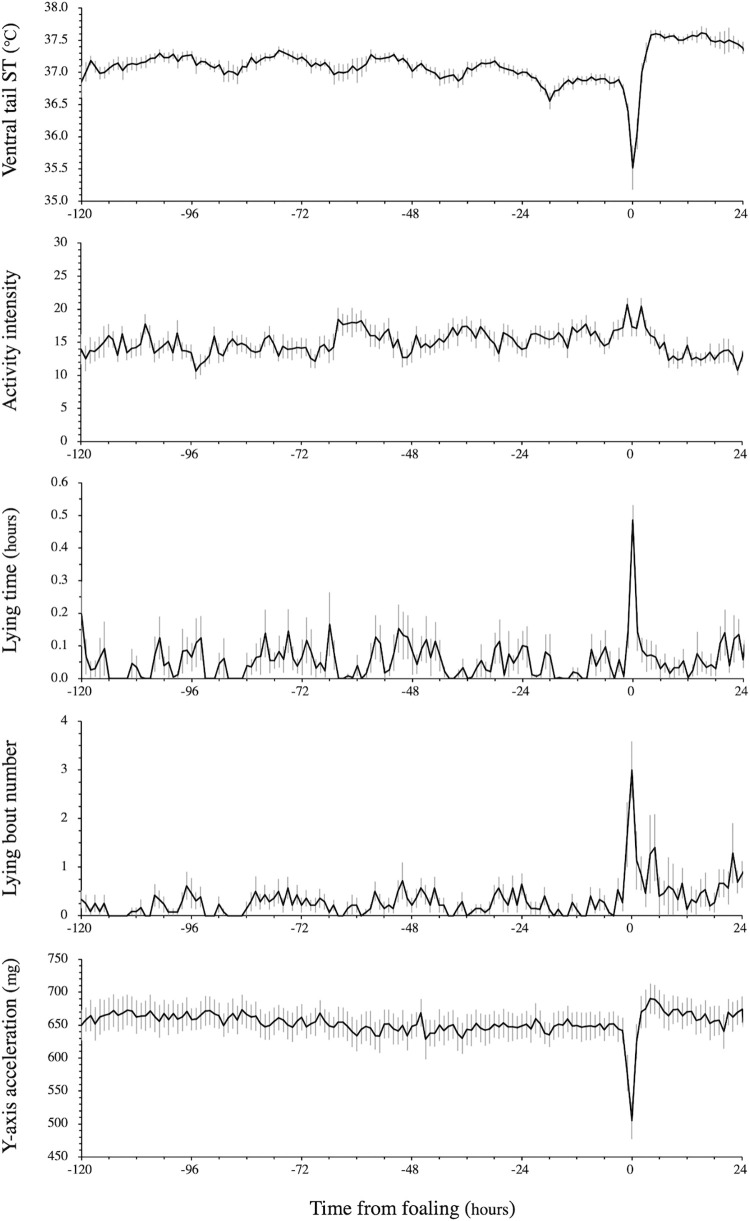
Changes in ventral tail ST, activity intensity, lying duration, and y-axis acceleration from 120 hours prepartum to 24 hours postpartum. The results were shown as mean ± standard error.

**Fig 5:**
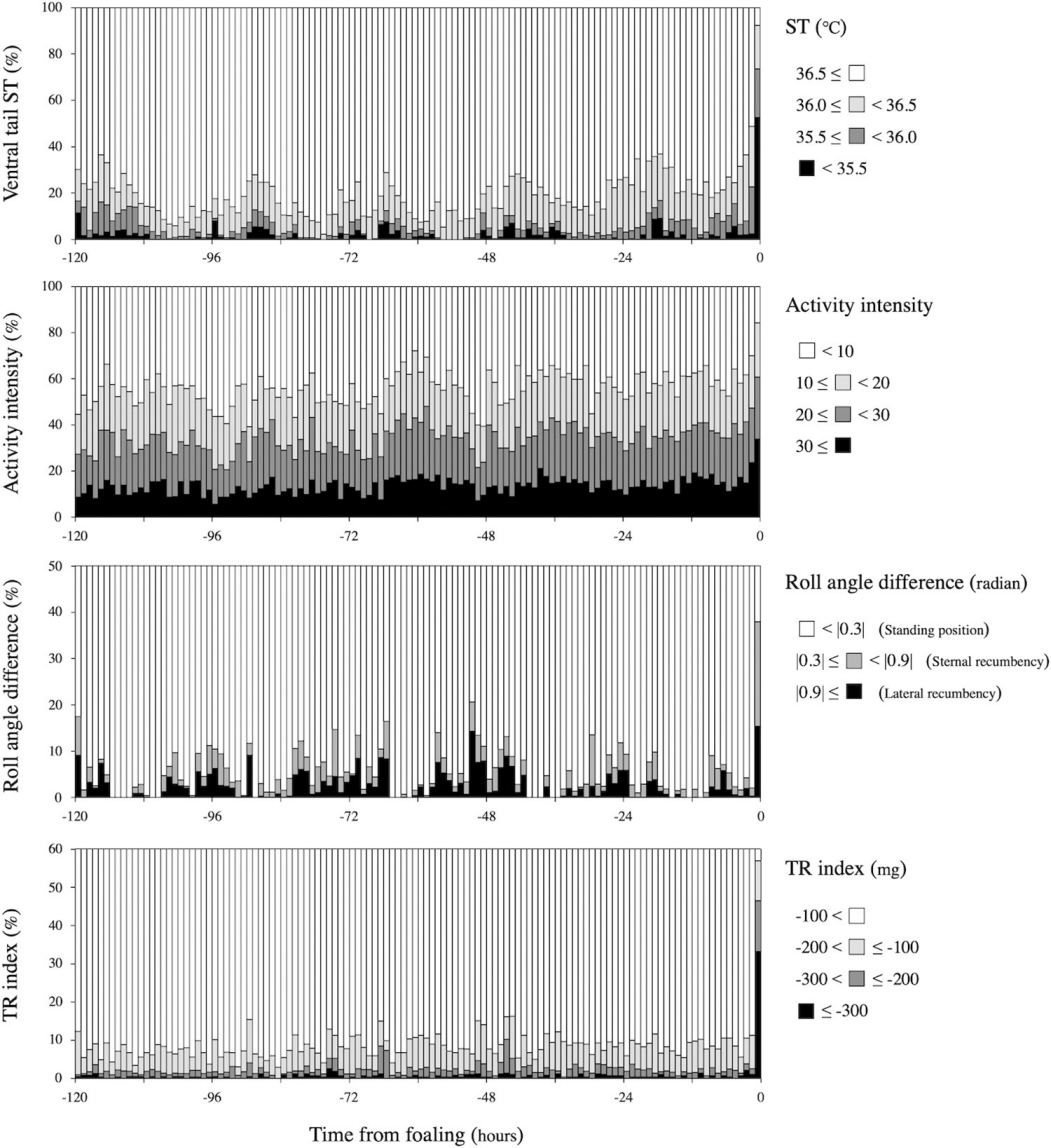
Changes in ventral tail ST, activity intensity, lying duration, and TR index for 120 hours before the time of birth of the foal. The data was classified by level in each parameter and shown as the occurrence rate per hour.

Hourly values for each parameter were extracted as follows: maximum values in ST, mean values in activity intensity, total time in lying posture, total number of lying bouts, mean values in y-axis acceleration. The results for these parameters are shown as mean ± standard error in **Fig 4**. The data was also classified by level in each parameter and is shown as the occurrence rate per hour in **Fig 5**.

The comparison of sensor data between 1 hour and 2−120 hours before delivery is shown in **Table 2**. The ventral tail ST sharply declined at 1 hour before foaling, with the percentage of sensor data having an ST below 35.5°C that was significantly higher at 1 hour than for 2−120 hours before foaling (pre-partum 1 hour vs. 2−120 hours; 52.5 vs. 1.7% per hour, P < 0.01). Activity intensity increased 1 hour before foaling with the percentage of sensor data (30 or more) being significantly higher at 1 hour than for 2−120 hours before the time of birth of the foal (pre-partum 1 hour vs. 2−120 hours; 34.0 vs. 13.4% per hour, P < 0.01). Lying duration, represented by roll angle, changed at 1 hour before the time of birth of the foal with the percentage of recumbent posture (|0.3| radians or more) being significantly higher at 1 hour than for 2−120 hours before the time of birth of the foal (pre-partum 1 hour vs. 2−120 hours; 38.0 vs. 5.0% per hour, P < 0.01). TR behavior changed at 1 hour before the time of birth of the foal with the percentage TR (-200 mg or less) being significantly higher at 1 hour than for 2−120 hours before the time of birth of the foal (pre-partum 1 hour vs. 2−120 hours; 46.5 vs. 2.3% per hour, P < 0.01).

**Table 2:** Hourly occurrence rate of physiological or behavioral parameters related to the time of birth of the foal (prepartum 1 hour vs. 2−120 hours).

Sensor data		Explanation	Hourly occurrence rate (%)					P-value (χ^2^ test)
			1 hour	2−120 hours				
				average	median	minimum	maximum	
ST	< 35.5°C	Lower ST	52.5	1.7	0.8	0	11.4	< 0.01
Activity intensity	≥ 30	Restlessness	34	13.4	13.5	5.7	23.7	< 0.01
Roll angle difference	≥ |0.3| radian	Recumbent posture	38	5	3.6	0	20.6	< 0.01
TR index	≤ -200 mg	TR behavior	46.5	2.3	2	0	10.3	< 0.01

ST indicates surface temperature of the ventral tail skin. TR index indicates a variation of y-axis acceleration from a reference value to a minus direction.

**Fig 6:**
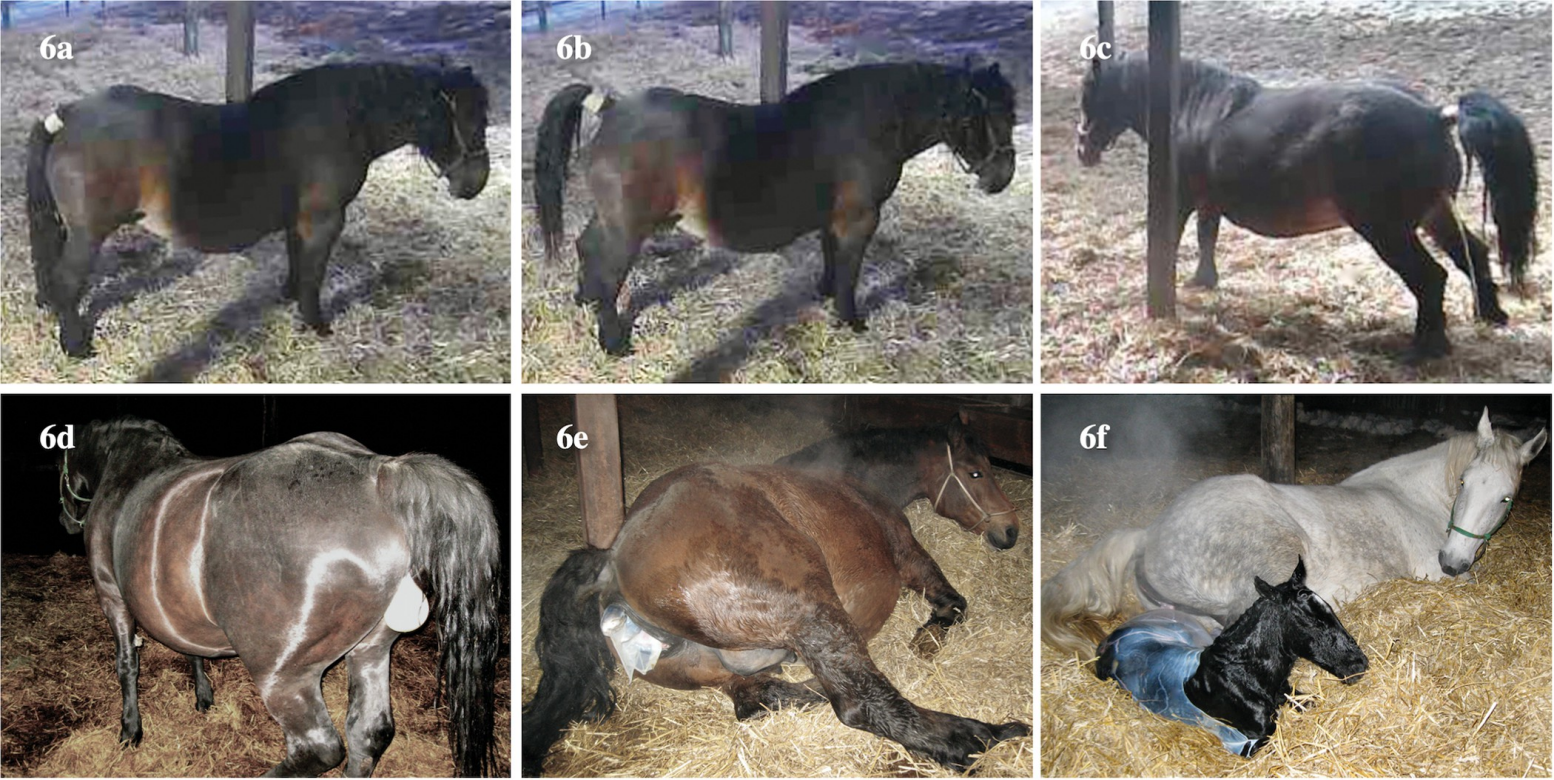
**6a**: This mare’s tail was in its usual position (not raising the tail). **6b**: This mare raised her tail when defecating. **6c**: This mare raised her tail when urinating. **6d**: Persistent TR can often be seen when the fetal sac (amniotic membrane containing amniotic fluid) comes out from the vulva. **6e**: Persistent TR can also be seen when the fetal foot comes out from the vulva. **6f**: Persistent TR may continue until the fetus is completely delivered. The photos used in Fig 6**d**-**6f** were taken in the other studies.

The video data was checked for 834 time points where the y-axis acceleration data decreased by -200 mg or less from the reference value (average value for 24 hours after the onset of recording), and found 623 time points that confirmed the TR behavior. The reasons why we could not confirm the mare’s behavior were: lack of video data; the horse being too far away from the camera to be seen; or the image being too dark to see the horse at night. Of the 623 time points, 51.8% (n = 323) were TR related to urination or defecation, and 23.0% (n = 143) were TR related to foaling (**Fig 6**). Also, TRs were observed while showing recumbent posture (19.1%, n = 119) and when wagging their tails to ward off insects (6.1%, n = 38).

Based on these results, three physiological or behavioral indices that showed a sharp increase in incidence just before parturition were determined as follows: (1) Lower ST (LST) below 35.5°C; (2) Lying down (LD), which is the change from standing to recumbent position (roll angle change from the reference value by ± 0.3 radian or more); (3) TR, which is the elevation of the tail from its usual position (y-axis acceleration decrease by 200 mg or more from the reference value). Using one or a combination of two or three of these indices, the possibility of delivery occurring within 1 hour after meeting the conditions was examined (**Table 3**). When the conditions of LST, LD and TR were analyzed alone, the sensitivity was 100% for all of them, but the precision was 13.1%, 8.1% and 2.8%, respectively. When two indices were combined, the sensitivity was 100% for all combinations, and the accuracies were 100% for the combination of LST and LD, 56.7% for the combination of LD and TR, and 32.1% for the combination of TR and LST. Sensitivity and precision were both 100% when the three conditions were combined. **Table 4** shows the time from meeting the indices (2 or 3) to the end of fetal delivery during the last hour prepartum. The average time to complete labor after two or more indices were met was 20−30 minutes.

**Table 3:** Probability of detecting the time of birth of the foal using three parameters.

Physiological or behavioral parameter			True positive	False positive	False negative	Sensitivity	Precision
**a**	**b**	**c**	**n**	**n**	**n**	**%**	**%**
LST			17	113	0	100	13.1
LD			17	193	0	100	8.1
TR			17	585	0	100	2.8
LST	LD		17	0	0	100	100
LD	TR		17	13	0	100	56.7
TR	LST		17	36	0	100	32.1
LST	LD	TR	17	0	0	100	100

LST was defined as the ventral tail ST being below 35.5°C in this study. LD was defined as a time point where standing posture changed to the recumbent posture, expressed by change of |0.3| radian or more from the reference values of roll angle. TR was defined as the decline over 200 mg from the reference values of y-axis acceleration. Time differences among parameter a, b and c were within 3 minutes.

**Table 4:** The time from when two or more conditions were observed in sensor data to the end of fetal delivery.

No.	Horse name	The time from when two or more conditions were observed in sensor data to the end of fetal delivery (minutes:seconds).			
		LST + LD	LD + TR	TR + LST	LST + LD + TR
1	VIO	08:46	08:46	20:45	08:46
2	KAN	08:40	11:40	08:40	08:40
3	GOL	33:37	15:36	54:37	15:36
4	SMI	20:02	23:02	20:02	20:02
5	SER	09:52	09:52	09:52	09:52
6	DEE	34:42	34:42	40:42	34:42
7	TOR	14:07	14:07	14:07	14:07
8	POW	18:46	21:46	18:46	18:46
9	BLU	43:25	43:25	46:25	43:25
10	WHI	08:11	08:11	14:11	08:11
11	YUN	46:35	16:34	19:34	16:34
12	YOS	12:12	12:12	12:12	12:12
13	RAS	51:26	51:26	51:26	51:26
14	SHO	36:13	36:13	45:13	36:13
15	KUR	22:47	22:47	28:47	22:47
16	TEI	42:29	42:29	42:29	42:29
17	HAM	21:34	21:34	21:34	21:34
**Average**		25:30	23:12	27:37	22:40
**Median**		21:34	21:34	20:45	18:46
**Minimum**		08:11	08:11	08:40	08:11
**Maximum**		51:26	51:26	54:37	51:26

LST was defined as the ventral tail ST being below 35.5°C in this study. LD was defined as a time point where standing posture changed to the recumbent posture, expressed by change of |0.3| radian or more from the reference values of roll angle. TR was defined as the decline over 200 mg from the reference values of y-axis acceleration. Time differences among three parameters were within 3 min.

## 4. Discussion

This present study detected the changes in ST and some behavioral parameters using a multimodal device with a thermistor and tri-axial accelerometer attached to the ventral tail base of the mare from 5 days prepartum to 1 day postpartum. Also, a few combinations of physiological or behavioral indicators have been shown to be useful for detecting the time of birth of the foal.

Previous studies have reported that core body temperature decreased in foaling mares [10, 12, 19]. We found that ventral tail base ST also decreased before foaling. Although progesterone has been shown to affect core body temperature in several animal species [26–28], the detailed relationship between endocrinological changes and the sharply decreasing body temperature that occurs just before delivery remains unclear. A recent study reported that skin temperature on the lateral side of the chest increased just before parturition in mares [29]. In this present study, we did not observe a rise in temperature in foaling mares. We found that ventral tail base ST may decrease regardless of the occurrence of foaling in our study, and we considered that this decrease in body temperature is mainly related to lying behavior. In the standing position, the sensor was pressed against the skin between the tail and the anus. LD possibly creates a gap between the tail and the anus, thus slightly separating the sensor from the skin, which in turn makes it susceptible to the effects of environmental temperature. Therefore, when trying to detect labor by combining data from the mare’s lying behavior with the change in ST, it is necessary to exclude a LST when it is followed by LD. Since the time difference between the two parameters was set to within 3 minutes in this present study, a LST caused by LD was excluded. Prolonged lying posture possibly leads to the cooling of the device attached to the ventral tail base. Therefore, the sharp decrease in ST observed during delivery in this present study might be due not only to the decrease in core body temperature, but also to the repeated and/or prolonged lying behaviors related to labor. In order to clarify the decrease in ST prior to the fetal delivery, it will be necessary to examine the ventral tail base ST using an infrared thermography camera. Body temperature is known to have a circadian rhythm of approximately 24 hours in the horse, with the lowest value at the early morning [30]. Future research should concentrate on identifying more detailed changes in ST around parturition by excluding the effects of diurnal variation. More foaling cases would be necessary to solve the problem. In a study that measured the ventral tail base ST using a wearable sensor, it was reported that the ST was affected by the environmental temperature [31]. Since the present study was conducted in a specific region and in a limited season, it was assumed that there was not a significant impact because of environmental conditions. It would be necessary to consider the effects of the environment in order to generalize the results obtained in this present study when applying them to horses around the world.

Our study found that standing and recumbent positions can be distinguished with very high reliability by using the changes in roll angle from the reference value. Of the 27,473 time points that could be visually confirmed by video, 25,978 (94.6%) were numerically determined as standing by the roll angle data. Of these 25,978 time points, 25,970 (99.9%) were confirmed that the mares were standing in the video. These results showed that the horses spent most of the day in a standing position. In rare cases, there were mares whose hips were barely tilted even in a sternal position, and these mares were judged not to be lying, but to be standing, based on the numerical values of roll angle. Of the 1,495 time points indicating a lying position (including sternal and lateral recumbency) as numerically determined from the sensor data, 20 (1.3%) were not actually lying based on video recordings. Prey animals such as horses typically sleep less than their predators. Horses sleep for approximately 12% of the day [32]. Schuurman et al. (2003) had reported that horses spend much of their life standing, and they are believed to be able to keep their limbs straight with little muscular effort [33]. Also, horses can drowse and sleep while standing, by means of the unique passive stay apparatus of the equine forelimbs and hindlimbs. These enable them to be on their feet for long periods of time with minimum muscular effort [34]. However, some effort must be required, since when the horse tires after a few minutes, it will shift its weight to the other hindlimb [34]. This behavior, when the horse shifts its weight onto one leg and let the other leg rest, is called ‘stand rest’. They repeat this behavior every few minutes, alternating between their left and right limbs [35]. This stand rest behavior was confirmed by video on most moments where the sensor data determined that the mare was in a recumbent posture, even though it was standing (**S1 File**). It is suggested that the stand rest behavior makes the buttock descend on the resting side, which affected the roll angle. It has been reported that lying behavior increases on the day of foaling when compared to the day before foaling [19]. To the best of our knowledge, there are no studies that analyze the behavior of mares on the day of foaling on an hourly basis. This present study has revealed that the lying duration and number of lying bouts increased significantly during the last hour before the foal is born, and that this rapid increase did not occur at 2 hours prepartum or before. In cattle and sheep, it has been reported that the number of lying bouts increased at about 4 to 6 hours before parturition [13, 17], which was thought to be restlessness as a maternal behavior. This present study suggests that mares only show restlessness when close to parturition, unlike other livestock and this may be related to the rapid progress of parturition in horses when compared to other farm animals. The second stage of parturition lasts only 15 to 20 minutes in mares [5]. The behavioral changes in the last hour before the time of birth of the foal also included behavioral changes related to labor. It will be necessary to clarify the relationship between the onset of second stage of labor and sensor data by observing the detailed foaling process.

In the present study, TR (based on a threshold of -200 mg from the reference value in y-axis acceleration) was most common during defecation and urination, followed by foaling-related TR. Horses defecate 6−12 times and urinate 3−6 times a day on average [36]. This means the sensor could be useful for detecting health problems such as less frequent defecation or urination. During fetal delivery, when the amniotic membrane emerges from the vulva, the tail is raised continuously until the fetus is expelled (**Fig 6D–6F**). Therefore, it has been shown that TR behavior with a large change in y-axis acceleration (decrease of 300 mg or more from the reference value) sharply increases at one hour before parturition (**Fig 5**). Also, since TR is more often observed in mares during the estrus period [37], it may be possible to apply this index to detect estrus.

We used a modified silicone rubber belt developed for cattle to attach the sensor to the tails of the mares. During the experimental period, 4 of the 17 mares had to have their sensors reattached, since they were inconsistent in recording body surface temperature. By the end of the experiment (a few days after foaling), none of the sensors or bands spontaneously dropped off. After removing the sensors, it was possible to observe in many mares the presence of scars at the sites of the skin where the devices were attached. By washing and applying an ointment for trauma, the scars healed in a few days. Since the examination of the mare’s milk during the prepartum can be useful for predicting time of foaling [38], adding this index can shorten the time that the device needs to be attached, reducing tail wounds. However, since it is often difficult to collect milk from nulliparous mares, it is expected that the device will have to be attached for a prolonged period in these mares. Improving the method of attachment of the sensor will be required when developing this technology for clinical application in the future.

In this present study, we used three physiological or behavioral indices, which rapidly increased just before fetal delivery, to verify the possibility of detecting the time of foaling. The LST (lower than 35.5°C), LD (changing from standing posture to recumbent posture, as determined by roll angle), and TR (decrease in y-axis acceleration of -200 mg or more from the reference value) had poor precision when used individually to detect foaling, being 13.1%, 8.1% and 2.8%, respectively. As described above, this was because of the influences of physiological behaviors such as LST associated with lying behavior, urination and defecation. Also, by combining these three indicators, we succeeded in improving the delivery detection accuracy. In particular, by combining LST and LD, or by combining all three indices, both sensitivity and precision achieved 100%, indicating the possibility that this method could be a useful tool for clinical application in the future.

The details associated with the onset of the second stage of labor such as rupture of allantochorion and/or appearance of the amnion outside the vulva could not be confirmed from the video recorded in most of the mares used in this present study. Rupture of the membrane usually occurs in the vagina and the amnion may not be visible on camera depending on the mare’s position. Therefore, it was not possible to clarify the relationship between the onset of labor and sensor data. Because the duration of second stage of labor is usually short in mares, recognizing the onset of foaling is very important for early detection and appropriate assistance for abnormal parturitions. It will be necessary to verify how quickly the onset of labor can be detected by the tail sensor before it is deemed appropriate for clinical application. Also, cases of dystocia such as abnormal fetal posture or significantly prolonged labor were not observed in this present study. The usefulness of the tail-attached sensor will have to be reconsidered after further examination of the effects of these abnormal foalings on sensor data.

## 5. Conclusion

In conclusion, the sequential changes in ST and in some behavioral parameters were identified using a multimodal device with a thermistor and tri-axial accelerometer attached to the ventral tail base of mares during their last 5 days of pregnancy. Also, it has been clarified that the time of birth of the foal can be detected with high sensitivity and precision by combining LST, LD, and TR. In the future, by increasing the number of cases, we would like to proceed with a more detailed perinatal behavioral analysis.

## Supporting information

S1 Data Raw data obtained from the sensors. (XLSX)

S1 File Stand rest behavior confirmed by video in this present study (ten-fold speed reproduction). (MP4)
